# Integrated genetic analysis reveals synaptic m^6^A enrichment in high-risk autism genes

**DOI:** 10.3389/fnmol.2026.1767983

**Published:** 2026-03-20

**Authors:** Shreya Doijad, Divyadharshini Sakthivel, Naveen Kumar Chandappa Gowda

**Affiliations:** 1 SKAN Research Trust, Bangalore, Karnataka, India; 2 Centre for Advance Research and Excellence in Autism and Developmental Disorders (CARE-ADD), St. John’s Research Institute, St. John’s National Academy of Health Science, Bangalore, Karnataka, India

**Keywords:** autism spectrum disorder, epitranscriptome, m^6^A, neurodevelopment, RNA, synaptopathy, translation regulation

## Abstract

Autism Spectrum Disorder (ASD), a complex neurodevelopmental condition, is characterised by reduced social and emotional expression and repetitive patterns of behaviour. The clinical observations of defects in brain development and disrupted connectivity in ASD correlate with the perturbations at the neuronal and molecular levels. While the underlying genetic basis has been extensively studied, understanding the epigenetic and epitranscriptomic regulation has only begun to unravel in the past two decades. This work aims to link the ASD clinical phenotypes to the molecular dysfunction, specifically highlighting one of the crucial mRNA modifications, N6-methyladenosine (m^6^A). During neuronal development, m^6^A, a key post-transcriptional regulator, dynamically modulates mRNA translation at synapses and is essential for maintaining synaptic plasticity. However, the mechanisms by which m^6^A operates at synapses in the context of ASD are poorly understood. Our work establishes connections across neuronal developmental timelines to m^6^A regulation and discusses the possibility of how this dysregulation may underlie the development of synaptopathies observed in ASD. By integrating previously published m^6^A-seq and CLIP-seq data with the SFARI gene database, we found that 41.59% (515 of 1,238 genes) of ASD risk genes are m^6^A-enriched. Specifically, we found 28 syndromic genes overlapping with the Synaptic m^6^A Epitranscriptome (SME). Here, we also shed light on the importance of m^6^A readers, with a focus on FMRP and YTHDF1 and their regulation at the synapse. Altogether, we suggest a model in which m^6^A-mediated post-transcriptional regulation influences ASD-related synaptic dysfunction.

## Graphical abstract

**Figure:**
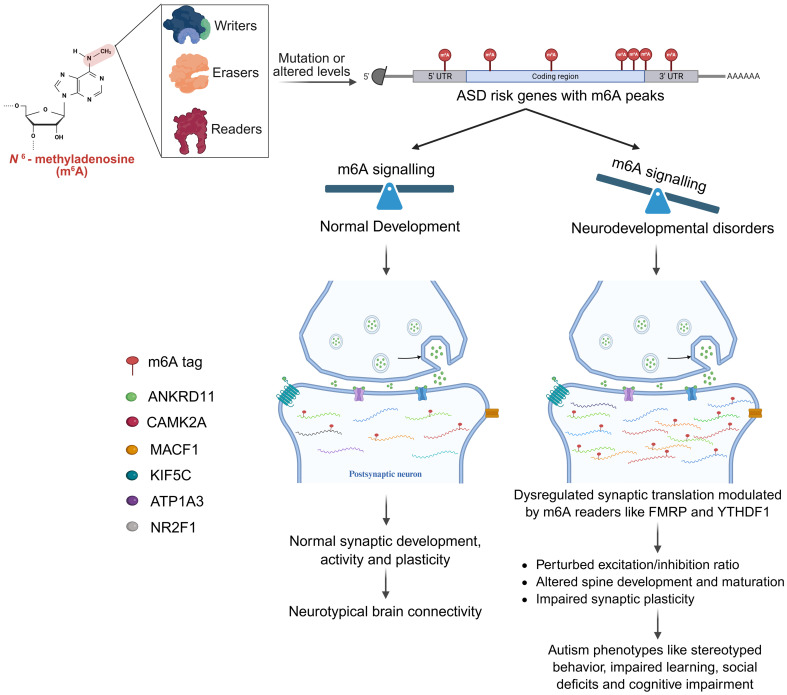

## Introduction

Autism is a multifaceted neurodevelopmental condition that widely impacts how a person perceives, processes, and responds to the world around them. According to the reports from the National Institute of Mental Health (NIMH) and the World Health Organisation (WHO), approximately 1 in 100 children worldwide are diagnosed with Autism (Zeidan et al., 2022). Autism is described as a “spectrum” disorder because it manifests in a wide range of symptoms and levels of severity.

While Autism Spectrum Disorder (ASD) is largely heritable due to genetic predisposition, several other risk factors, including epigenetic and environmental factors, influence its development (Hallmayer et al., 2011; Cheroni et al., 2020). Symptoms of ASD majorly include social interaction impairment, delayed language development, communication difficulties and repetitive behaviours (Baird and Norbury, 2016; Berry et al., 2018). Given the considerable clinical heterogeneity among individuals with autism, the recent version of DSM-5 (Diagnostics and Statistical Manual-5) of the American Psychiatric Association has revised and expanded the diagnostic concept and criteria (Regier et al., 2013). Numerous genes have been implicated in regulating the co-occurring features of ASD, and many of these genes are found to be inherited variations (Srividhya et al., 2024). However, precise mechanisms by which these genetic alterations lead to the hallmark features of ASD remain partially understood. Along with the ASD risk genes, prenatal environmental factors like hypoxia and alterations in the neuro-epigenome also contribute to ASD (Eshraghi et al., 2018). This includes alterations in DNA methylation and transcriptome modifications (Tran et al., 2019; Tseng et al., 2022; Shafik et al., 2022).

The majority of the RNA species undergo dynamic modifications, tagged with diverse chemical moieties, called Epitranscriptomic modifications. They play a key role in maintaining cellular homeostasis by regulating RNA folding, transcription, splicing, stability, axonal transport, and translation (Wang et al., 2014; Yang et al., 2018). Among the many epitranscriptomic marks that regulate RNA fate, N6-methyladenosine (m^6^A) is the most abundant modification in eukaryotic RNAs, accounting for ~80% of total RNA base methylations and also the most abundant mRNA modifications in the brain and CNS (Chang et al., 2017; Chokkalla et al., 2020; Wang et al., 2021). Primarily occurring on the adenine nucleotide in the DRACH motif of the RNA sequence (D = A, G, or U; R = A or G; H = A, C, or U), its distribution varies across development and exhibits high tissue-specificity (Zhang et al., 2020).

To enable the function of m^6^A, it requires m^6^A methylation machinery, and it has 3 essential components, namely, Writers, Erasers, and Readers. The core mammalian methyltransferase complex (writers) comprises METTL3, METTL14 and WTAP, which catalyse targeted m^6^A deposition. This modification is reversible and can be removed by the demethylases FTO and ALKBH5. The m^6^A marks on the transcripts are recognised by a set of m^6^A-binding proteins called Readers. The functioning of m^6^A tags is mediated by these reader proteins, and they regulate their stability, translation, export, splicing and decay. Some of the important readers include YTH-domain containing proteins like YTHDC1/2, YTHDF1/2/3, and eIF3, FMRP and hnRNPA2/B1/C, which lack the YTH-domain.

A comprehensive study in 2012 uncovered the m^6^A distribution in mice brain using MERIP-Seq, and found about 13,471 m^6^A peaks in a total of 4,654 genes (Meyer et al., 2012). Of this, about 94.5% peaks were found in mRNAs and the remaining in several classes of ncRNAs. The mRNAs listed encoded genes involved in various brain regulatory functions such as ion channels, metabotropic receptors, transcription factors, cytoskeletal, axon guidance, synaptic plasticity, learning, and memory (Chokkalla et al., 2020; Widagdo et al., 2022). About 2,921 genes in adult mouse forebrain identified as synaptic m^6^A epitranscriptome (SME) are involved in the formation of tripartite synapses and in pathways seen in neurodevelopmental and psychiatric disorders (Merkurjeve al., 2018; Takumi et al., 2020). m^6^A modifications are detected throughout embryogenesis, with a drastic increase observed during adulthood in mice, implying that their upregulation is closely linked to neuronal maturation along with nervous system development (Meyer et al., 2012; Hess et al., 2013; Yoon et al., 2017).

The above studies indicate that m^6^A has emerged as a major post-transcriptional regulator, and although the functional significance of m^6^A in gene regulation has been extensively characterised in recent years, its specific involvement in neurodevelopmental disorders (NDDs), particularly ASD, remains insufficiently understood. To address this gap, we investigated whether a substantial proportion of ASD-associated genes are preferentially marked by m^6^A during early neurodevelopmental events like neurogenesis and synaptogenesis.

Autism is often referred to as a synaptopathy, as numerous ASD risk genes encode proteins that either localise to and function at synapses (Guang et al., 2018). Majorly, these proteins regulate multiple aspects of synaptic functions, such as cell adhesion and synapse formation (Neurexins, Neuroligins, Cadherins), Scaffolding (SHANK, PTEN, PSD-95), as well as maturation and protein synthesis regulators (SYNGAP1, FMR1). Modulation of local mRNA translation mechanisms in the cell body and dendritic spine contributes to synaptic abnormalities in ASD (Takumi et al., 2020). In this study, we also investigate a plausible role of m^6^A mRNA transcripts and suggest a model in which m^6^A-mediated post-transcriptional regulation influences ASD-related synaptic dysfunction.

## Results

### Correlation of m^6^A methylation to brain development till birth

**Figure 1:**
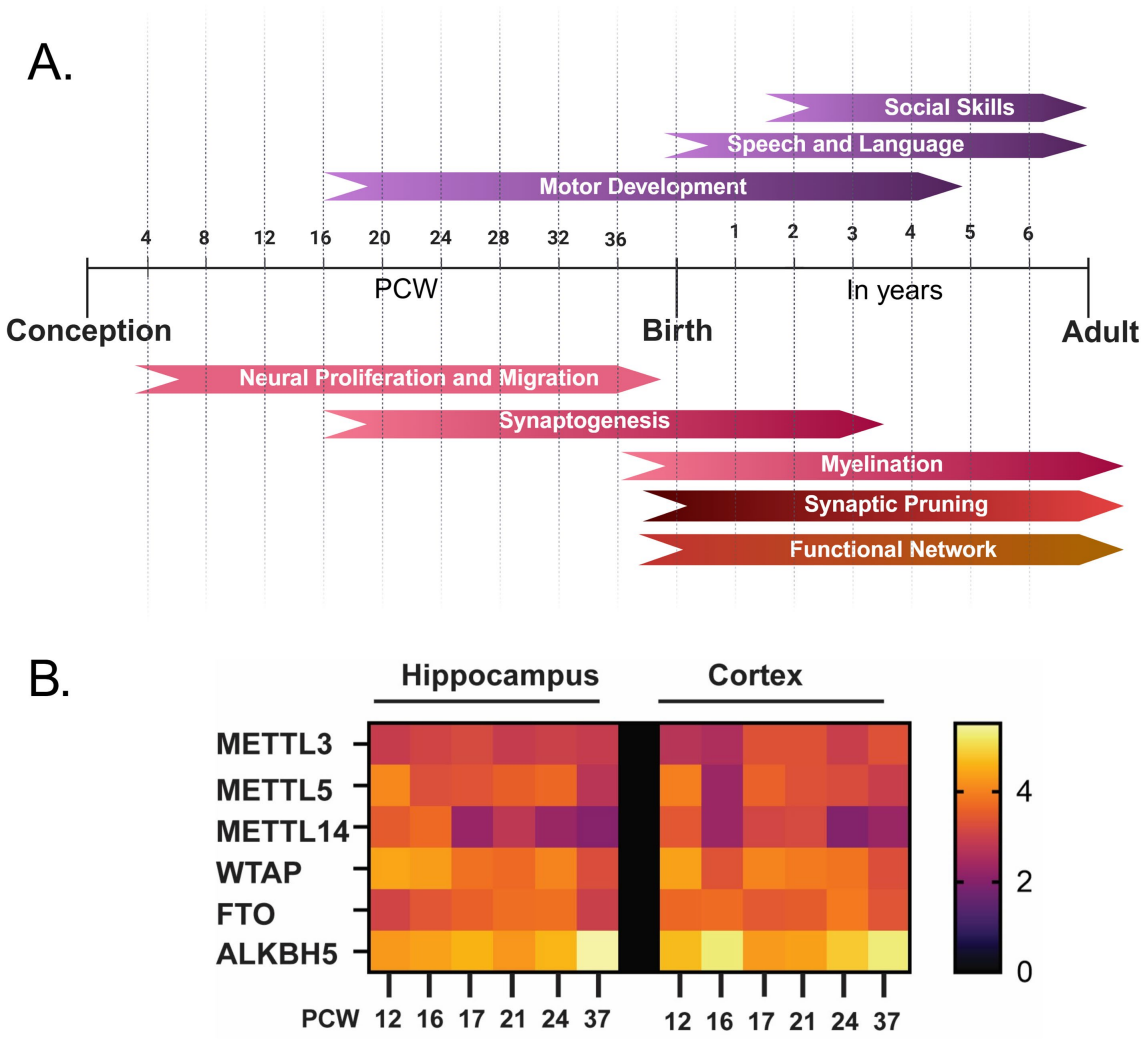
Correlation of m^6^A-associated protein levels to brain development. **(A)** Timeline for normal brain development – from conception to infancy. **(B)** Heatmap representing the expression levels of m^6^A writers (METTL3, METTL14, METTL5, and WTAP) and erasers (FTO and ALKBH5) in hippocampus and cortex during human fetal development (PCW 12–PCW 37) as reported in Allen BrainSpan Atlas (Kang et al., 2011).

Typically, brain development begins as early as post-conception week (PCW) 4 and developmental events, starting from neuronal proliferation till functional network development events are indicated in (Figure 1A). NDDs result from disruptions in the highly coordinated events essential for proper brain development. In ASD, there is a widespread dysregulation across multiple stages of early brain development. Numerous studies have indicated a prenatal origin of molecular and cellular defects underlying the behavioural deficits observed in ASD. This includes enlarged brain size due to a 67% increase in neuron number in the prefrontal cortex (Courchesne et al., 2011), neurogenesis and neuronal migration defects (Gilbert and Man, 2017; Courchesne et al., 2019). Furthermore, ASD risk genes substantially converge in the development of the cortex between PCW 8 to 24 (Courchesne et al., 2019). It is thought that the multigenic state of ASD arises from epigenetic effects on multiple genes.

We know that neurogenesis is a dynamic process, and we were interested in understanding the nature of these proteins during neurogenesis. For this, we used data from the Allen BrainSpan Atlas, which indicates that the expression and activity of the methylation machinery vary across developmental time and brain regions. Interestingly, out of all m^6^A regulatory proteins, we found that METTL14 expression decreases, whereas ALKBH5 increases, in the cortex and hippocampus during embryonic development (PCW 12–PCW 37) (Figure 1B). These shifts coincide with critical periods of neurogenesis, synaptogenesis, and synaptic pruning (Yoon et al., 2017; Martinez De La Cruz et al., 2021). m^6^A regulation is more active during embryonic stages than in the postnatal stage, where transcripts encoding transcription factors and neurogenesis regulators are frequently methylated (Zhang et al., 2020; Shao et al., 2023). This facilitates rapid turnover and tight temporal control of gene expression. Alteration in the expression levels of these regulators modulates transcript methylation and expression patterns during brain development (Supplementary Table 1). For instance, the METTL14 knockout model exhibits impaired radial glial cell differentiation and delayed generation of cortical neuronal subtypes. This is mainly caused by the failure in m^6^A deposition by the methytransferase complex. These observations clearly link dysregulated m^6^A dynamics to neurodevelopmental disorders (NDD) (Chokkalla et al., 2020).

### SFARI ASD risk genes exhibit a high prevalence of m^6^A-methylation

To investigate whether m^6^A regulates genes implicated in ASD, we integrated the SFARI Gene database (Simons Foundation Autism Research Initiative; https://gene.sfari.org/), which encompasses all the syndromic and non-syndromic ASD-implicated genes. The critical period of early brain development in humans, which includes neural proliferation and differentiation, begins as early as PCW 4 and remains highly active until PCW 20.

**Figure 2:**
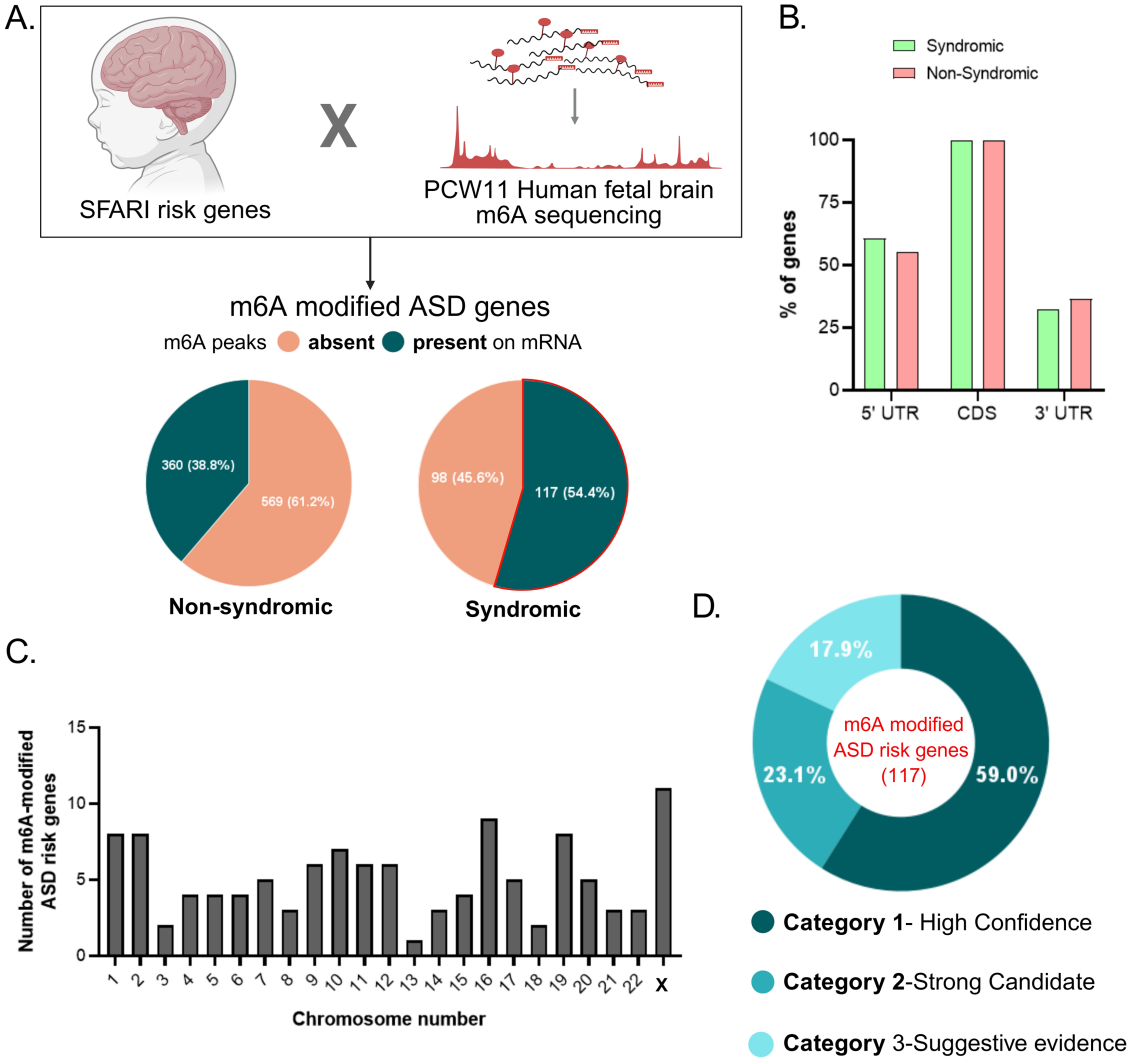
Prevalence of m^6^A-modified genes in ASD. **(A)** Segregation of SFARI ASD genes by their m^6^A modification status from human fetal brain m^6^A-seq data. Blue: m^6^A modification present on mRNA; Orange: m^6^A modification absent on mRNA in both syndromic and non-syndromic SFARI genes. **(B)** Distribution of m^6^A peaks on the ASD mRNA transcripts, syndromic (green) and non-syndromic (red), at PCW 11. **(C)** Histogram distribution of m^6^A ASD risk genes (syndromic) across human chromosomes. *X*-axis indicating chromosome number and *Y*-axis indicating the number of m^6^A-modified ASD risk genes per chromosome. **(D)** Classification of m^6^A-modified ASD risk genes (syndromic) into category 1, 2, and 3 by severity score provided by SFARI. SFARI Gene: An evolving database for the autism research community. Simons Foundation Autism Research Initiative. Available at: https://gene.sfari.org. (2026).

Previously, Yoon et al. (2017) generated a m^6^A-sequencing database for PCW 11 human fetal brain, providing m^6^A mRNA profiles during human cortical neurogenesis. Using this dataset, we quantified the proportion of SFARI genes carrying m^6^A modifications at PCW 11. A total of 515 out of 1,238 SFARI genes (41.59%) exhibited m^6^A enrichment. Next, we assessed the non-syndromic genes and found that 360 of 969 genes (38.7%) were m^6^A-modified. Whereas, 117 of 215 syndromic genes (54.42%) were m^6^A-modified (Supplementary Table 2; Figure 2A), indicating a comparatively higher prevalence of m^6^A tagging in syndromic ASD genes.

Further, we were interested in understanding the distribution of m^6^A across syndromic and non-syndromic transcripts. Analysis of m^6^A distribution along the mRNA transcripts suggested that all 515 genes displayed m^6^A within exonic regions. Additionally, 55 and 36.6% of non-syndromic genes found m^6^A within the 5′UTR and 3′UTR, respectively (Figure 2B). The data revealed a similar pattern between both categories. Syndromic genes showed a comparable distribution, with 60.7 and 32.5% carrying m^6^A in the 5′UTR and 3′UTR, respectively (Figure 2B). Interestingly, this data is consistent with a previous report, demonstrating that there is a higher m^6^A proportion in CDS regions of fetal tissues compared to adult tissues (Zhang et al., 2020). Thus, the observed m^6^A-profile has a specific role during neurogenesis and changes over the developmental timeline (Figure 2B).

To understand the distribution of syndromic m^6^A-modified ASD risk genes (117) across the chromosomes, we performed chromosomal distribution analysis. This revealed that the X chromosome harbours the highest number of 11 ASD genes carrying m^6^A methylation. Notably, key genes including FMR1, MECP2, PCDH19, and FGF13 were enriched for m^6^A methylation (Figure 2C). Upon further stratification of these genes according to the SFARI Gene scoring system, a substantial proportion, 59% of the m^6^A-modified syndromic genes, were observed within Category 1 (High Confidence) (Figure 2D). The remaining genes were distributed across Category 2 (23.1%; Strong Candidate) and Category 3 (17.9%; Suggestive Evidence) (Figure 2D).

Apart from the SFARI syndromic genes, several other non-syndromic gene candidates strongly implicated in ASD also undergo m^6^A modifications (Figure 2A). Some of the Category 1 genes include CTNNB1, NRXN2, NRXN1, GRIN1 and MAP1A.

Further, we looked into how CTNNB1, the gene encoding *β*-catenin, is identified as a Category 1 gene in the SFARI database. The m^6^A peaks were found in the coding sequence of its mRNA, which is regulated by m^6^A writer METTL3. CTNNB1 forms a transcriptional cascade with Brn2/Tbr2 prenatally and is essential for adult social behaviours (Tang et al., 2021). It has been reported that neural progenitor cells (NPCs) derived from skin fibroblasts of ASD patients showed increased cell proliferation due to dysregulated CTNNB1/Brn2 transcriptional cascade. These changes have contributed to the ASD behavioural phenotypes seen in Dishevelled mutants (Dvl1 and Dvl3) (Belinson et al., 2016).

Overall, our observations support that m^6^A acts as an additional post-transcriptional regulator for ASD linked genes during early development.

### m^6^A enrichment in syndromic ASD correlates with synaptic defects

**Figure 3:**
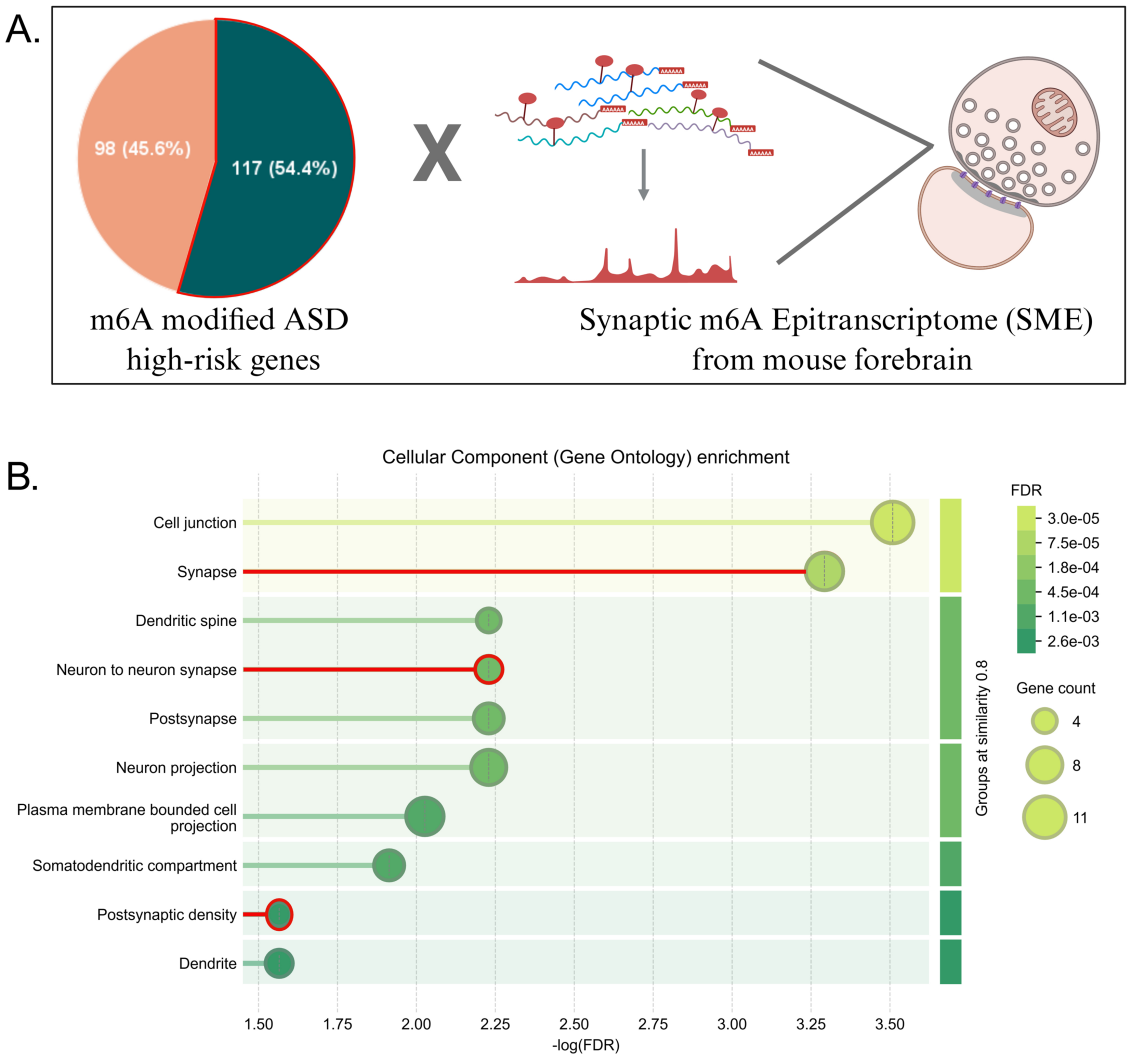
M^6^A-modified ASD high-risk genes associated with the synaptopathology. **(A)** Analysis of m^6^A-modified ASD risk genes overlapped with mouse forebrain SME, indicating ASD genes enriched for synaptic functions. **(B)** Gene ontology enrichment analysis performed using STRING for cellular components on 17 out of 28 genes exhibiting cytoplasmic localisation. The components highlighted in red represent terms that were also enriched in PAN-GO analysis.

Previous reports suggest that active translation occurs at the synapse, and defects in mRNA regulation by m^6^A modification in ASD are not well understood. For this, we used a mouse forebrain synaptic m^6^A dataset (SME) (Merkurjeve al., 2018). We took a similar approach where we analysed the 117 m^6^A-modified ASD risk genes overlapping with the SME (Figure 3A). We identified 28 of the 117 genes within the SME dataset, suggesting these candidates may undergo m^6^A-mediated regulation at synapses and thereby impact synaptic function. We have summarised the role of these 28 genes and their role in neurodevelopment (Table 1). Notably, out of 28 genes, nine were predominantly nuclear, including transcription factors and chromatin remodelling proteins enriched in the nBAF and SWI/SNF complexes. As their roles within the synaptic compartment remain poorly characterised, these genes were excluded from the Gene Ontology (GO) analysis. GO enrichment analysis for cellular components revealed significant enrichment in cell junction, synapse, dendritic spine, neuron projection, alongside others (Figure 3B). This was independently validated by an alternative tool (PAN-GO), which identified the enriched cellular components (FDR *p*-value < 0.05) to be postsynaptic density, asymmetric synapse, neuron-to-neuron synapse, postsynaptic specialisation and synapse. The terms common to both analyses are highlighted in red in Figure 3B. All of these genes are actively expressed in the cortex during neurogenesis as well as synaptogenesis (Supplementary Table 2). The key proteins within the interaction network include CAMK2A, PTEN, SHANK3, IQSEC2, ANKS1B and SLC6A1. Mutations in these genes are associated with synaptic dysfunction and the behavioural phenotype seen in ASD and Epilepsy (Roy et al., 2020). This data strongly suggests the importance of m^6^A methylation on these mRNA candidates, possibly controlling the mRNA localisation or stability, impacting on synaptic function in ASD.

**Table 1:** m^6^A ASD high-risk genes associated with the synaptopathology.

Serial number	Gene symbol	Gene name	SFARI gene score	Human brain development process involved
1.	ANKRD11	Ankyrin repeat domain 11	1	Neurogenesis, Dendritic growth
2.	ANKRD17*	Ankyrin repeat domain 17	2	Neuronal maturation
3.	ANKS1B	Ankyrin repeat and sterile alpha motif domain containing 1B	2	Synaptogenesis, synaptic plasticity
4.	ARID1A*	AT-rich interaction domain 1A	3	Neuronal proliferation and differentiation
5.	ATP1A3	ATPase Na+/K + transporting subunit alpha 3	2	Synaptic plasticity
6.	CAMK2A	Calcium/calmodulin-dependent protein kinase II alpha	1	Dendritic spine organisation, synaptic plasticity
7.	CDK19	Cyclin-dependent kinase 19	3	Synaptogenesis
8.	CHD3*	Chromodomain helicase DNA binding protein 3	1	Neuronal migration, Axon guidance
9.	CHD7*	Chromodomain helicase DNA binding protein 7	1	Neural proliferation
10.	DNMT3A*	DNA (cytosine-5-)-methyltransferase 3 alpha	1	Neuronal maturation, Synaptic plasticity
11.	EHMT1*	Euchromatic histone-lysine N-methyltransferase 1	1	Neurogenesis and neuronal maturation
12.	IQSEC2	IQ motif and Sec7 domain 2	1	Dendritic spine and axonal growth
13.	KDM3B*	Lysine demethylase 3B	1	Neuronal maturation
14.	KIF5C	Kinesin family member 5C	2	Synaptogenesis, neuronal migration
15.	MACF1	Microtubule actin crosslinking factor 1	3	Axon guidance and neuronal migration
16.	NACC1	Nucleus accumbens associated 1	1	Synaptic plasticity
17.	NFIB*	Nuclear factor I B	2	Neurogenesis
18.	NR2F1	Nuclear receptor subfamily 2 group F member 1	2	Neuronal differentiation and migration
19.	PPFIA3	PTPRF interacting protein alpha 3	3	Synaptogenesis and vesicle release
20.	PRR12	Proline-rich 12	1	Insufficient literature
21.	PTEN	Phosphatase and tensin homolog	1	Axon guidance, Neuronal migration
22.	SHANK3	SH3 and multiple ankyrin repeat domains 3	1	Neuronal maturation, synaptic plasticity
23.	SLC6A1	Solute carrier family 6 (neurotransmitter transporter), member 1	1	Neurotransmission
24.	SMARCC2*	SWI/SNF related, matrix associated, actin-dependent regulator of chromatin, subfamily c, member 2	1	Neuronal proliferation and differentiation
25.	TCF20*	Transcription factor 20 (AR1)	1	Neuronal differentiation
26.	TRIM8	Tripartite motif containing 8	3	Insufficient literature
27.	UGGT1	UDP-glucose glycoprotein glucosyltransferase 1	3	Insufficient literature
28.	ZFHX3*	Zinc finger homeobox 3	3	Neuronal proliferation and differentiation

*Primary nuclear localisation.

### Regulation of m^6^A-modified high-risk ASD genes at synapse by FMRP and YTHDF1

Having identified a subset of m^6^A-modified ASD risk genes associated with synaptic function, we next examined whether these transcripts are recognised by m^6^A readers that regulate mRNA translation. We examined two well-characterised m^6^A readers, FMRP and YTHDF1, both known to mediate m^6^A-dependent regulation of mRNA translation in neurons and along axons (Zhang et al., 2018; Broix et al., 2025).

**Figure 4:**
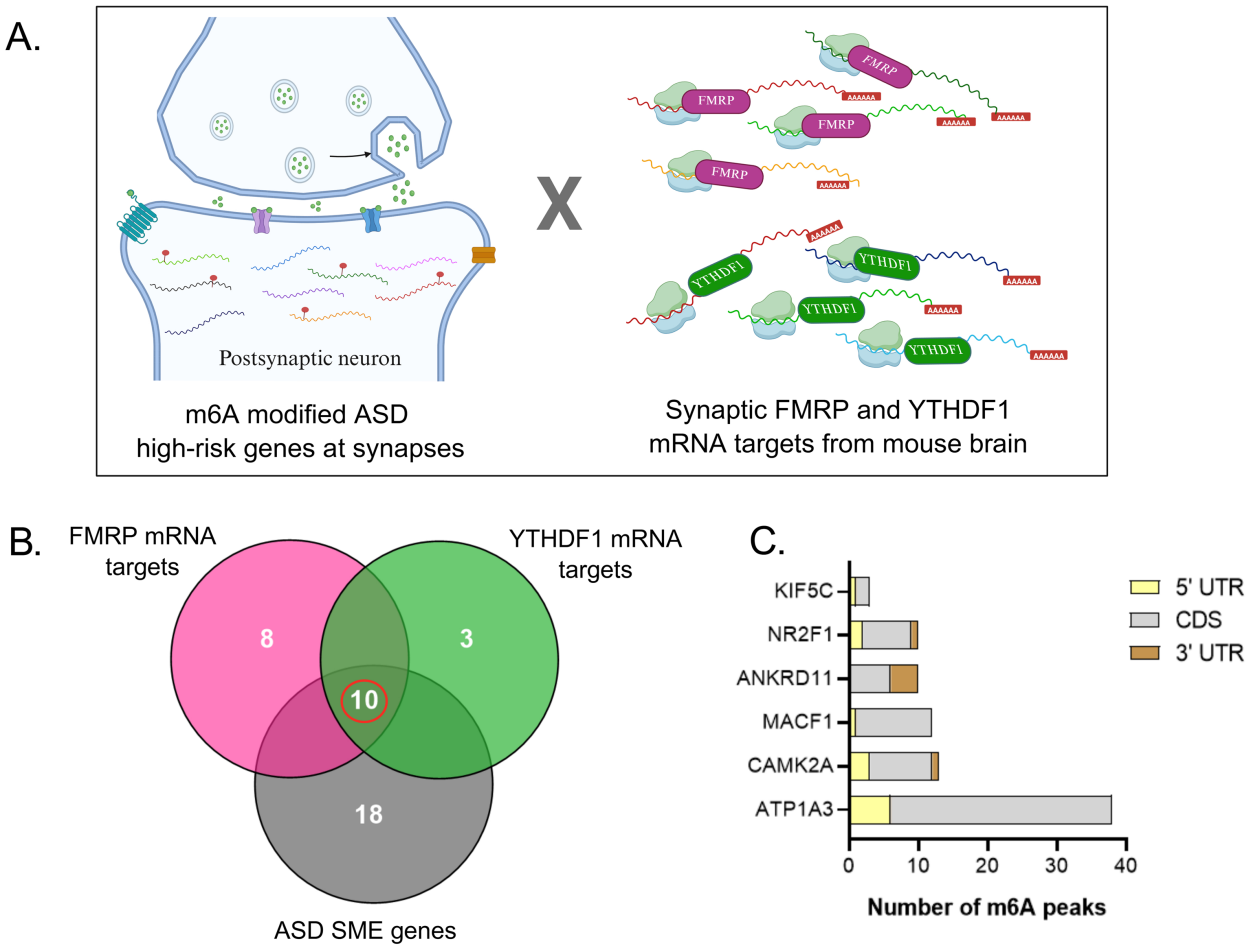
Synaptic FMRP and YTHDF1 regulate m^6^A methylation of ASD high-risk genes. **(A)** Subset of overlapping m^6^A-modified ASD high-risk genes at synapses with known FMRP (pink) and YTHDF1 (green) targets in neurons. **(B)** Venn diagram illustrating the overlap among mRNA targets of FMRP (pink), mRNA targets of YTHDF1 (green), and ASD SME genes (grey). A total of 10 genes are common to all three datasets, as highlighted in the center. **(C)** Distribution of m^6^A peaks across transcript regions of six candidate mRNA targets shared by FMRP and YTHDF1.

We used published CLIP-seq data for FMRP and YTHDF1 (Darnell et al., 2011; Shi et al., 2018) and assessed their overlap with the 28 m^6^A-modified ASD risk genes at synapse (Figure 4A). We found that 18 genes are established neuronal targets of FMRP and 13 are targets of YTHDF1 (Figure 4B). Strikingly, ten of these genes were common to both datasets, supporting the possibility of co-regulation by both reader proteins. The shared subset of synapse localised high-risk genes included CAMK2A, ANKRD11, MACF1, ATP1A3, KIF5C and NR2F1. We excluded the genes that are primarily nuclear and are not shown to localise to the cytoplasm (ANKRD17, ARID1A, SMARCC2 and TCF20). We examined the distribution of m^6^A across the transcript length (5’UTR, CDS, 3’UTR) for six genes using the PCW11 m^6^A-sequencing database. Primarily, we observed that m^6^A sites were enriched within the CDS region for all the genes, indicating dynamic regulation of these mature mRNA candidates during translation. As discussed previously, this observation corroborates prior data showing CDS-prevalent m^6^A distribution in the embryonic brain. KIF5C showed overall low m^6^A modification and a lack of m^6^A in the 3′ UTR. Whereas, ATP1A3 showed strong CDS bias (32 peaks), with comparatively fewer peaks in the 5′ UTR (6 peaks) and a complete absence in the 3′ UTR. Overall, we observed a non-uniform and gene-specific distribution of m^6^A peaks across these regions (Figure 4C).

Further, we discuss one of the candidate genes, CAMK2A, with a particular focus on its regulation and contribution to the ASD phenotype through plausible m^6^A regulation. CAMK2A, which encodes the *α*-subunit of Ca^2+^/calmodulin-dependent protein kinase 2, is involved in the stabilisation of dendritic protrusions, essential for synaptic plasticity and memory (Jia et al., 2014). CAMK2A was identified as a target of YTHDF1 and carries distinct m^6^A peaks (Shi et al., 2018). The modification occur in both exonic and intronic regions and is prominently enriched in the CDS according to the PCW 11 m^6^A sequencing data (9 peaks). A *de novo* Glu183Val (E183V) variant in CAMK2A was identified in a patient with ASD, and expression of this mutant in hippocampal neurons disrupted dendritic morphology and synaptic transmission. Homozygous E183V mutant mice showed a significant disruption in CAMK2A interactions, displayed enhanced repetitive behaviours and deficits in social interactions (Stephenson et al., 2017). In addition to this, it interacts with proteins such as Shank3, GluN2B, and mGlu5 that regulate synaptic functions and are strongly linked to ASD. Overall, these data suggested the dynamic role of m^6^A methylation at the synapse in both syndromic and non-syndromic candidates, and further experimental validations are needed on our observations to provide clinical relevance.

## Discussion

Multiple studies have suggested that defects in ASD are linked to m^6^A regulation, yet no concise reports are available with an integrated approach. Here, we provide a systemic analysis of ASD high-risk genes demonstrating that the modulation of m^6^A modification on them would contribute to associated synaptic defects.

A total of 10,980 high-confidence m^6^A methylation peaks are associated with 5,049 transcripts in the human PCW 11 fetal brain. This accounts for approximately 31.4% of detected transcripts at that developmental stage (Yoon et al., 2017). Here, we demonstrate that m^6^A is prevalent on about 41.59% of the total SFARI dataset (515 of 1,238 genes), which includes both the syndromic and non-syndromic ASD associated genes. Additionally, a substantial proportion of Syndromic SFARI genes are m^6^A-regulated. The predominance of Category 1 genes among the m^6^A-modified set indicates that transcripts with the strongest and most reproducible genetic association to ASD are preferentially marked by m^6^A during early cortical development, particularly neurogenesis (Figures 2A,D). This enrichment suggests that m^6^A-dependent post-transcriptional regulation may be particularly relevant for high-confidence ASD risk genes, reinforcing the potential contribution of epitranscriptomic mechanisms to ASD pathogenesis. Furthermore, a notable enrichment, especially on the X chromosome, suggests m^6^A may disproportionately modulate transcripts that confer sex-biased vulnerability (2.8% in males and 0.65% in females) (Loomes et al., 2017) (Figure 2C). This also provides a basis for the underlying molecular mechanism in X-linked inheritance patterns observed in several syndromic forms of ASD, such as Fragile X Syndrome (FXS), Rett syndrome, and AUTSX variants.

Extending this analysis to the synaptic compartment, we identified a subset of m^6^A-modified ASD risk genes enriched for synaptic and postsynaptic components. Also, the majority of m^6^A components regulate the biological processes in the early developmental window and converge in synapse regulation pathways, indicating a crosstalk between them (Supplementary Table 1). A significant fraction of these transcripts are targets of both the m^6^A readers FMRP and YTHDF1 in the neurons (Supplementary Table 2). In the present study, we report a shared subset of ten high-risk genes, co-targeted by both m^6^A readers within the synaptic compartment, where m^6^A-dependent translational control could directly contribute to synaptic dysfunctions implicated in ASD (Figure 4B,C). This subset includes ANKRD11, ATP1A3, CAMK2A, KIF5C, MACF1 and NR2F1. We have excluded the four candidates which do not show reported synaptic localisation. However, evidence is emerging that supports the presence of traditionally nuclear proteins (TFs and chromatin modifiers) within axonal compartments, where they may have non-canonical functions. Therefore, some of the excluded candidates may also prove to have m^6^A-regulated synaptic roles. Additionally, the non-uniform m^6^A distribution across the six selected candidate genes highlights distinct regulatory roles for m^6^A sites in CDS versus UTRs (Figure 4C).

Neurons, as highly specialised and polarised cells, depend on local protein synthesis to maintain synaptic plasticity. It allows rapid change in the synaptic proteome upon stimulation by receptors such as metabotropic Glutamate Receptors (mGluRs) and N-methyl-D-Aspartate Receptors (NMDARs). Several studies have indicated an imbalance in local protein translation in ASD and FXS (Kobayashi et al., 2017; Feuge et al., 2019). In axons and at synapses, m^6^A methylation status on the mRNA can act as a reversible switch that can fine-tune the mRNA levels and regulate its translation in response to stimulation (Yu et al., 2018). It may have opposite effects on local translation, wherein it promotes translation in dendrites but inhibits in axons (Livneh et al., 2020). Various brain regions, including the hippocampus and prefrontal cortex, exhibit dynamic regulation of m^6^A upon sensory or behavioural stimuli (Walters et al., 2017). There is significant upregulation of m^6^A levels in a subset of transcripts in the prefrontal cortex during behavioural training in mice (Walters et al., 2017). Similarly, an increased m^6^A methylation was observed in the dorsal hippocampus following fear conditioning in mice (Quan et al., 2021). These studies strongly indicate that the m^6^A deposition is responsive to neuronal activity and behavioural experience. This consistent increase in m^6^A levels has been primarily attributed to decreased expression of FTO (Yen and Chen, 2021; Leonetti et al., 2024). Supporting this mechanism, a study revealed that stimulation of the NMDA receptor in rat primary neurons significantly reduced FTO levels, thereby increasing the m^6^A levels and triggering global translation inhibition (Gowda et al., 2022).

YTHDF1 enhances protein synthesis of m^6^A-modified mRNAs in mouse hippocampal neurons in a stimulus-dependent manner (Shi et al., 2018). It also interacts with FMRP, which is a translational repressor, alongside being an m^6^A reader. Under basal conditions, unphosphorylated FMRP associates with YTHDF1 and limits its ability to promote translation. However, upon KCl stimulation (which mimics an mGluR antagonist), FMRP becomes phosphorylated, leading to dissociation of the FMRP–YTHDF1 complex, which enables translation of YTHDF1 targets. Therefore, in addition to FMRP regulating translation of its own mRNA targets, it indirectly controls m6A-mediated translation via YTHDF1. As supported by our observations, the subset of high-risk ASD genes co-targeted by both FMRP and YTHDF1 could be particularly noteworthy underlying ASD synaptopathy. Altered regulation of either of these m^6^A readers or the m^6^A profiles on their transcripts can impair synaptic functions. In a mouse model of FXS, a known monogenic cause of ASD, a significant increase in YTHDF1-dependent m^6^A mRNA translation in neurons was shown, further contributing to disease progression in FXS (Zou et al., 2023). This finding also strongly indicates that there is dysregulation in m^6^A-mediated activity-dependent protein synthesis in FXS.

Together, our findings from the integration of the genomic databases signify m^6^A as a promising epitranscriptomic layer regulating ASD risk genes across early brain development and synaptic function. Even though there is substantial progress in the field of post-transcriptional modifications, very few studies show the activity-dependent regulation of m^6^A-tagged transcripts in the developing brain, especially at the synapses. Moving forward, focused mapping of m^6^A at cell-type and subcellular resolution during the prenatal windows of brain development, combined with functional perturbation of candidate m^6^A targets, will be essential to determine whether m^6^A alterations drive ASD pathogenesis.

## Method

### Data source

All analyses were performed using publicly available datasets from previously published studies. The human fetal brain m^6^A-sequencing dataset was obtained from Yoon et al. (2017). The data were accessed from the GEO accession number GSE99017, with sample accession GSM2742603. The mouse forebrain synaptic m^6^A profiling dataset was retrieved from Supplementary Table 5 of Merkurjev et al., 2018. The corresponding m^6^A-sequencing data is available under accession number PRJNA388019.

FMRP CLIP-seq data were obtained from Supplementary Table 2 of Darnell et al., 2011, with GEO accession number GSE26809. YTHDF1 CLIP-seq target data were retrieved from Supplementary Table 2 of Shi et al., 2018, with GEO accession number GSE106607.

The SFARI Gene database (Simons Foundation Autism Research Initiative; https://gene.sfari.org/) was used to retrieve required information about the syndromic and non-syndromic genes and their categorisation. Gene expression data were obtained from the Allen BrainSpan Atlas of the Developing Human Brain for selected brain regions between PCW 12 and PCW 37 (https://www.brainspan.org/).

### Overlap analysis

Gene overlap between two datasets was assessed using LibreOffice 7.2.2.2. Gene symbols from one list were compared against the second list using the MATCH function in Excel. Specifically, the formula = MATCH (cell_reference, range, 0) was applied, where the target gene symbol was searched against the comparison gene list. The non-matching genes returned an error value (#N/A), whereas the shared genes returning a numeric value were considered overlapping entries. This was filtered to remove the non-matching gene entries.

### Functional annotation

The GO Analysis for cellular components enrichment was carried out for 17 genes from Table 1 using the PAN-GO Human Functionome Version 2.0 and the STRING web tools independently. Fisher’s exact test with FDR correction was used to identify significant enrichment, *p* < 0.05. Functional enrichment visualisation and protein interaction network analysis were carried out using STRING, and terms were grouped at default similarity > = 0.8. Genes marked with * in Table 1 were excluded from this analysis.

## References

[ref1] Baird G. Norbury C. F. (2016). Social (pragmatic) communication disorders and autism spectrum disorder. Arch. Dis. Child. 101, 745–751. doi: 10.1136/archdischild-2014-306944, 26699538

[ref2] Belinson H. Nakatani J. Babineau B. A. Birnbaum R. Y. Ellegood J. Bershteyn M. . (2016). Prenatal β-catenin/Brn2/Tbr2 transcriptional cascade regulates adult social and stereotypic behaviors. Mol. Psychiatry 21, 1417–1433. doi: 10.1038/mp.2015.207, 26830142 PMC5685528

[ref3] Berry K. Russell K. Frost K. (2018). Restricted and repetitive behaviors in autism spectrum disorder: a review of associated features and presentation across clinical populations. Curr. Dev. Disord. Rep. 5, 108–115. doi: 10.1007/s40474-018-0139-0

[ref4] Broix L. Roy R. Shohayeb B. Oomoto I. Umeshima H. Wang D. O. (2025). m6A RNA methylation-mediated control of global APC expression is required for local translation of β-actin and axon development. Cell Rep. 44:115727. doi: 10.1016/j.celrep.2025.115727, 40402742

[ref5] Chang M. Lv H. Zhang W. Ma C. He X. Zhao S. . (2017). Region-specific RNA m6A methylation represents a new layer of control in the gene regulatory network in the mouse brain. Open Biol. 7:7170166. doi: 10.1098/rsob.170166 PMC5627058 28931651

[ref6] Cheroni C. Caporale N. Testa G. (2020). Autism spectrum disorder at thecrossroad between genes and environment: contributions, convergences, and interactions in ASD developmental pathophysiology. Mol. Autism. 11:69. doi: 10.1186/s13229-020-00370-1, 32912338 PMC7488083

[ref7] Chokkalla A. K. Mehta S. L. Vemuganti R. (2020). Epitranscriptomic regulation by m6A RNA methylation in brain development and diseases. J. Cereb. Blood Flow Metab. 40, 2331–2349. doi: 10.1177/0271678X20960033, 32967524 PMC7820693

[ref8] Courchesne E. Mouton P. R. Calhoun M. E. Semendeferi K. Ahrens-Barbeau C. Hallet M. J. . (2011). Neuronnumber and size in prefrontal cortex of children with autism. JAMA 306, 2001–2010. doi: 10.1001/jama.2011.1638, 22068992

[ref9] Courchesne E. Pramparo T. Gazestani V. H. Lombardo M. V. Pierce K. Lewis N. E. (2019). The ASD living biology: from cell proliferation to clinical phenotype. Mol. Psychiatry 24, 88–107. doi: 10.1038/s41380-018-0056-y, 29934544 PMC6309606

[ref10] Darnell J. C. Van Driesche S. J. Zhang C. Hung K. Y. Mele A. Fraser C. E. . (2011). FMRP stalls ribosomal translocation on mRNAs linked to synaptic function and autism. Cell 146, 247–261. doi: 10.1016/j.cell.2011.06.013, 21784246 PMC3232425

[ref11] Eshraghi A. A. Liu G. Kay S. S. Eshraghi R. S. Mittal J. Moshiree B. . (2018). Epigenetics and autism spectrum disorder: is there a correlation? Front. Cell. Neurosci. 12:78. doi: 10.3389/fncel.2018.00078 29636664 PMC5881102

[ref12] Feuge J. Scharkowski F. Michaelsen-Preusse K. Korte M. (2019). FMRP modulates activity-dependent spine plasticity by binding Cofilin1 mRNA and regulating localisation and local translation. Cereb. Cortex 29, 5204–5216. doi: 10.1093/cercor/bhz059, 30953439

[ref13] Gilbert J. Man H.-Y. (2017). Fundamental elements in autism: from neurogenesis and neurite growth to synaptic plasticity. Front. Cell. Neurosci. 11:359. doi: 10.3389/fncel.2017.00359, 29209173 PMC5701944

[ref14] Gowda N. K. C. Nawalpuri B. Ramakrishna S. Jhaveri V. Muddashetty R. S. (2022). NMDAR mediated dynamic changes in m6A inversely correlates with neuronal translation. Sci. Rep. 12:11317. doi: 10.1038/s41598-022-14798-3, 35790863 PMC9256623

[ref15] Guang S. Pang N. Deng X. Yang L. He F. Wu L. . (2018). Synaptopathology involved in autism Spectrum disorder. Front. Cell. Neurosci. 12:470. doi: 10.3389/fncel.2018.00470, 30627085 PMC6309163

[ref16] Hallmayer J. Cleveland S. Torres A. Phillips J. Cohen B. Torigoe T. . (2011). Genetic heritability and shared environmental factors among twin pairs with autism. Arch. Gen. Psychiatry 68, 1095–1102. doi: 10.1001/archgenpsychiatry.2011.76, 21727249 PMC4440679

[ref17] Hess M. E. Hess S. Meyer K. D. Verhagen L. A. Koch L. Brönneke H. S. . (2013). The fat mass and obesity associated gene (Fto) regulates activity of the dopaminergic midbrain circuitry. Nat. Neurosci. 16, 1042–1048. doi: 10.1038/nn.3449, 23817550

[ref001] Jia J. M. Hu Z. Nordman J. Li Z. (2014). The schizophrenia susceptibility gene dysbindin regulates dendritic spine dynamics. The Journal of neuroscience: the official journal of the Society for Neuroscience 34, 13725–13736. doi: 10.1523/JNEUROSCI.0184-14.2014 25297099 PMC4188970

[ref002] Kang H. J. Yuka I. K. Feng C. Ying Z. Xuming X. Mingfeng L. . (2011). “Spatio-temporal transcriptome of the human brain”. Nature, 478, 483–489. doi: 10.1038/nature10523 22031440 PMC3566780

[ref18] Kobayashi S. Tanaka T. Soeda Y. Almeida O. F. X. Takashima A. (2017). Local Somatodendritic translation and hyperphosphorylation of tau protein triggered by AMPA and NMDA receptor stimulation. EBioMedicine 20, 120–126. doi: 10.1016/j.ebiom.2017.05.012, 28566250 PMC5478209

[ref19] Leonetti A. M. Galluzzo I. R. McLean T. A. D. Stefanelli G. Ramnaraign F. Holm S. . (2024). The role of the m6A/m demethylase FTO in memory is both task and sex-dependent in mice. Neurobiol. Learn. Mem. 210:107903. doi: 10.1016/j.nlm.2024.107903, 38403011

[ref20] Livneh I. Moshitch-Moshkovitz S. Amariglio N. Rechavi G. Dominissini D. (2020). The m6A epitranscriptome: transcriptome plasticity in brain development and function. Nat. Rev. Neurosci. 21, 36–51. doi: 10.1038/s41583-019-0244-z, 31804615

[ref21] Loomes R. Hull L. Mandy W. P. L. (2017). What is the male-to-female ratio in autism Spectrum disorder? A systematic review and Meta-analysis. J. Am. Acad. Child Adolesc. Psychiatry 56, 466–474. doi: 10.1016/j.jaac.2017.03.013, 28545751

[ref22] Martinez De La Cruz B. Markus R. Malla S. Haig M. I. Gell C. Sang F. . (2021). Modifying the m6A brain methylome by ALKBH5-mediated demethylation: a new contender for synaptic tagging. Mol. Psychiatry 26, 7141–7153. doi: 10.1038/s41380-021-01282-z, 34663904 PMC8872986

[ref23] Merkurjev D. Hong W.-T. Iida K. Oomoto I. Goldie B. J. Yamaguti H. . (2018). Synaptic N6-methyladenosine (m6A) epitranscriptome reveals functional partitioning of localized transcripts. Nat. Neurosci. 21, 1004–1014. doi: 10.1038/s41593-018-0173-6, 29950670

[ref24] Meyer K. D. Saletore Y. Zumbo P. Elemento O. Mason C. E. Jaffrey S. R. (2012). Comprehensive analysis of mRNA methylation reveals enrichment in 3' UTRs and near stop codons. Cell 149, 1635–1646. doi: 10.1016/j.cell.2012.05.003, 22608085 PMC3383396

[ref25] Quan W. Li J. Liu L. Zhang Q. Qin Y. Pei X. . (2021). Influence of N6-Methyladenosine modification gene HNRNPC on cell phenotype in Parkinson’s disease. Parkinson's Dis. 2021:9919129. doi: 10.1155/2021/9919129, 34966539 PMC8712163

[ref26] Regier D. A. Kuhl E. A. Kupfer D. J. (2013). The DSM-5: classification and criteria changes. World Psychiatry 12, 92–98. doi: 10.1002/wps.20050, 23737408 PMC3683251

[ref27] Roy R. Li X. Hou S. Fujiwara Y. Sukegawa M. Hong W.-T. . (2020). Schizophrenia and autism associated mutations and disrupted m6A signal by YTHDF1 cause defects in microtubule function and neurodevelopment (p. 2020.11.14.382556). bioRxiv. doi: 10.1101/2020.11.14.382556

[ref28] Shafik A. M. Allen E. G. Jin P. (2022). Epitranscriptomic dynamics in brain development and disease. Mol. Psychiatry 27, 3633–3646. doi: 10.1038/s41380-022-01570-2, 35474104 PMC9596619

[ref29] Shao N. Ye T. Xuan W. Zhang M. Chen Q. Liu J. . (2023). The effects of N6-methyladenosine RNA methylation on the nervous system. Mol. Cell. Biochem. 478, 2657–2669. doi: 10.1007/s11010-023-04691-6, 36899139

[ref30] Shi H. Zhang X. Weng Y.-L. Lu Z. Liu Y. Lu Z. . (2018). M6A facilitates hippocampus-dependent learning and memory through YTHDF1. Nature 563, 249–253. doi: 10.1038/s41586-018-0666-1, 30401835 PMC6226095

[ref31] Srividhya D. Parambath S. V. Sathyanarayanan R. Huligerepura Sosalegowda A. Korlimarla A. Niranjana Murthy A. S. . (2024). Whole exome sequencing of a multiplex family of Indian origin identifies variants in the RAI1 and FLII genes within the 17p11.2 region in siblings with autism and Smith-Magenis syndrome. Molecular syndromology 15, 537–544. doi: 10.1159/000539400, 39634244 PMC11614432

[ref32] Stephenson J. R. Wang X. Perfitt T. L. Parrish W. P. Shonesy B. C. Marks C. R. . (2017). A novel human CAMK2A mutation disrupts dendritic morphology and synaptic transmission, and causes ASD-related behaviors. J. Neurosci. 37, 2216–2233. doi: 10.1523/JNEUROSCI.2068-16.2017, 28130356 PMC5338762

[ref33] Takumi T. Tamada K. Hatanaka F. Nakai N. Bolton P. F. (2020). Behavioral neuroscience of autism. Neurosci. Biobehav. Rev. 110, 60–76. doi: 10.1016/j.neubiorev.2019.04.012, 31059731

[ref34] Tang Y. Chen K. Song B. Ma J. Wu X. Xu Q. . (2021). m6A-atlas: a comprehensive knowledgebase for unraveling the N6-methyladenosine (m6A) epitranscriptome. Nucleic Acids Res. 49, D134–D143. doi: 10.1093/nar/gkaa692, 32821938 PMC7779050

[ref35] Tran S. S. Jun H. I. Bahn J. H. Azghadi A. Ramaswami G. Van Nostrand E. L. . (2019). Widespread RNA editing dysregulation in brains from autistic individuals. Nat. Neurosci. 22, 25–36. doi: 10.1038/s41593-018-0287-x, 30559470 PMC6375307

[ref36] Tseng C. J. McDougle C. J. Hooker J. M. Zürcher N. R. (2022). Epigenetics of autism Spectrum disorder: histone deacetylases. Biol. Psychiatry 91, 922–933. doi: 10.1016/j.biopsych.2021.11.021, 35120709

[ref37] Walters B. J. Mercaldo V. Gillon C. J. Yip M. Neve R. L. Boyce F. M. . (2017). The role of the RNA demethylase FTO (fat mass and obesity-associated) and mRNA methylation in hippocampal memory formation. Neuropsychopharmacology 42, 1502–1510. doi: 10.1038/npp.2017.31, 28205605 PMC5436121

[ref38] Wang X. Lu Z. Gomez A. Hon G. C. Yue Y. Han D. . (2014). N6-methyladenosine-dependent regulation of messenger RNA stability. Nature 505, 117–120. doi: 10.1038/nature12730, 24284625 PMC3877715

[ref39] Wang J. Sha Y. Sun T. (2021). M6A modifications play crucial roles in glial cell development and brain tumorigenesis. Front. Oncol. 11:611660. doi: 10.3389/fonc.2021.611660, 33718165 PMC7943831

[ref41] Widagdo J. Wong J. J. Anggono V. (2022). The m6A-epitranscriptome in brain plasticity, learning and memory. Semin. Cell Dev. Biol. 125, 110–121. doi: 10.1016/j.semcdb.2021.05.023, 34053866

[ref42] Yang Y. Hsu P. J. Chen Y.-S. Yang Y.-G. (2018). Dynamic transcriptomic m6A decoration: writers, erasers, readers and functions in RNA metabolism. Cell Res. 28, 616–624. doi: 10.1038/s41422-018-0040-8, 29789545 PMC5993786

[ref43] Yen Y.-P. Chen J.-A. (2021). The m6A epitranscriptome on neural development and degeneration. J. Biomed. Sci. 28:40. doi: 10.1186/s12929-021-00734-6, 34039354 PMC8157406

[ref44] Yoon K.-J. Ringeling F. R. Vissers C. Jacob F. Pokrass M. Jimenez-Cyrus D. . (2017). Temporal control of mammalian cortical neurogenesis by m6A methylation. Cell 171, 877–889.e17. doi: 10.1016/j.cell.2017.09.003, 28965759 PMC5679435

[ref45] Yu J. Chen M. Huang H. Zhu J. Song H. Zhu J. . (2018). Dynamic m6A modification regulates local translation of mRNA in axons. Nucleic Acids Res. 46, 1412–1423. doi: 10.1093/nar/gkx1182, 29186567 PMC5815124

[ref46] Zeidan J. Fombonne E. Scorah J. Ibrahim A. Durkin M. S. Saxena S. . (2022). Global prevalence of autism: a systematic review update. Autism Res 15, 778–790. doi: 10.1002/aur.2696, 35238171 PMC9310578

[ref47] Zhang L. Du K. Wang J. Nie Y. Lee T. Sun T. (2020). Unique and specific m6A RNA methylation in mouse embryonic and postnatal cerebral cortices. Genes 11:1139. doi: 10.3390/genes11101139, 32992647 PMC7650744

[ref48] Zhang F. Kang Y. Wang M. Li Y. Xu T. Yang W. . (2018). Fragile X mental retardation protein modulates the stability of its m6A-marked messenger RNA targets. Hum. Mol. Genet. 27, 3936–3950. doi: 10.1093/hmg/ddy292, 30107516 PMC6216232

[ref49] Zhang H. Shi X. Huang T. Zhao X. Chen W. Gu N. . (2020). Dynamic landscape and evolution of m6A methylation in human. Nucleic Acids Res. 48, 6251–6264. doi: 10.1093/nar/gkaa347, 32406913 PMC7293016

[ref51] Zou Z. Wei J. Chen Y. Kang Y. Shi H. Yang F. . (2023). FMRP phosphorylation modulates neuronal translation through YTHDF1. Mol. Cell 83, 4304–4317.e8. doi: 10.1016/j.molcel.2023.10.028, 37949069 PMC10872974

